# Bridging mechanism and clinic: unlocking the full potential of oncolytic virus-based immunotherapy

**DOI:** 10.1186/s12943-026-02651-4

**Published:** 2026-03-31

**Authors:** Xinru Hua, Hengyi Tang, Kaijie Liang, Jian Li, Xiaotian Zhang, Lin Shen, Cheng Zhang

**Affiliations:** 1 Department of Gastrointestinal Oncology, State Key Laboratory of Holistic Integrative Management of Gastrointestinal Cancers, Beijing Key Laboratory of Carcinogenesis and Translational Research, Peking University Cancer Hospital & Institute, Fu-Cheng Road 52, Hai-Dian District, 100142 Beijing, China; 2 Department of Gastrointestinal Oncology, Key laboratory of Carcinogenesis and Translational Research (Ministry of Education/Beijing), Peking University Cancer Hospital & Institute, 52 Fucheng Road, Beijing, 100142 China; 3 Department of Gastrointestinal Oncology, Peking University Cancer Hospital (Inner Mongolia Campus)/Affiliated Cancer Hospital of Inner Mongolia Medical University, Hohhot, China

**Keywords:** Oncolytic viruses, Immunotherapy, Tumor microenvironment, Targeted delivery, Clinical trials, Combination therapy

## Abstract

Oncolytic viruses (OVs) have emerged as a distinctive class of cancer therapeutics capable of coupling direct tumor cell lysis with broad immune activation, thereby reshaping antitumor immunity beyond the capacities of conventional modalities. Despite substantial progress, the clinical translation of OVs remains constrained by challenges such as inefficient delivery, restricted tumor selectivity, premature immune clearance and safety concerns, all of which collectively limit therapeutic efficacy. Recent advances in genetic engineering, viral retargeting and high-dimensional profiling technologies have begun to clarify the molecular and microenvironmental determinants of OV tropism and activity, providing new opportunities to optimize OV design.

In this review, we synthesize current OV classifications, mechanisms of action and clinical developments, and highlight emerging innovations spanning synthetic engineering, targeted delivery platforms and combination therapy strategies—including those integrating OVs with immunotherapies, targeted agents and conventional modalities—to amplify therapeutic potency and overcome resistance. Together, these perspectives provide an integrated framework for understanding OV biology, underscore the need to address persistent barriers to safe and effective clinical deployment, and outline key priorities for advancing OVs toward their potential as a foundational component of next-generation, personalized cancer immunotherapy.

## Introduction

Cancer continues to be a major cause of mortality worldwide [1–3]. Multidisciplinary efforts are advancing cancer treatment [4], and conventional therapeutic modalities—including surgical resection, chemotherapy, radiotherapy, and evidence-based combination therapies—have shown progressive improvements in cure rates and long-term survival outcomes [5–7]. However, achieving complete eradication of malignancies remains clinically challenging due to factors such as tumor heterogeneity and acquired treatment resistance [8, 9]. Consequently, novel therapeutic strategies are needed to overcome these limitations [10–12].

In this context, Cell and Gene Therapy (CGT) is an innovative therapeutic approach targeting diseases at the molecular and cellular levels [13, 14]. Unlike traditional anticancer drugs, which primarily inhibit tumor growth, CGT intervenes directly in the molecular mechanisms of disease by introducing functional genes or engineered cells to achieve precise, long-lasting, and potentially curative effects. Clinical evidence shows CGT is rapidly unveiling its therapeutic potential in oncology, paving the way for truly personalized and precise cancer therapy [15–17].

Among CGT, OVs are a particularly promising strategy. These viruses selectively replicate within and lyse tumor cells while sparing healthy tissues. Beyond direct oncolytic effects, OVs induce immunogenic cell death(ICD) and enhance antitumor immunity [18]. Since the mid-19th century, numerous clinical cases have documented tumor regression coinciding with natural viral infections [19]. A landmark achievement occurred in 2015 when the U.S. Food and Drug Administration (FDA) approved Talimogene laherparepvec (T-VEC) a genetically modified oncolytic herpes simplex virus type 1 (oHSV-1), as the first OV therapy for treating metastatic melanoma patients [20]. To date, four oncolytic virus therapies have received global regulatory approval: H101, T-VEC, ECHO-7a, and Teserpaturev [21, 22].

Notably, early 2025 saw two groundbreaking Chinese advances in oncolytic virotherapy. A team at the First Affiliated Hospital of Zhejiang University School of Medicine developed the oncolytic virus VG161 and reported its Phase I clinical trial results. Their multicenter Phase I study represents the first clinical evaluation of VG161’s safety, pharmacokinetics, and preliminary efficacy in patients with refractory hepatocellular carcinoma. This novel OV, engineered to carry four immune-modulating genes, demonstrated not only an excellent safety profile but also the remarkable ability to reactivate the host immune system against drug-resistant tumors [23].

In another significant breakthrough, a research team from Guangxi Medical University developed an intravenous OV therapy based on a recombinant Newcastle disease virus carrying the porcine α1,3-galactosyltransferase (α1,3GT) gene (NDV-GT). This innovative approach achieved remarkable clinical success in advanced cancer treatment. Upon intravenous administration, NDV-GT reprograms tumor cells to express α-gal epitopes, effectively “camouflaging” them as porcine xenografts. This triggers a potent hyperacute rejection response, tricking the immune system into attacking the tumors and resulting in tumor growth inhibition or even complete eradication [24].

Despite this progress, OV therapy still faces several critical challenges. Administration routes—intratumoral and intravenous—exhibit significant limitations : intratumoral injection is constrained by tumor accessibility, while intravenous administration faces systemic clearance and pre-existing host antiviral immunity [25]. Consequently, there is an urgent need to develop novel delivery systems to enhance tumor targeting and reduce off-target risks [26]. Furthermore, precisely modulating the balance between antiviral immune responses and antitumor immunity represents another pivotal scientific challenge. Core bottlenecks include replication restrictions and toxicity risks (cytokine release syndrome (CRS), neurotoxicity [27, 28]). Particularly, reliable biomarker systems capable of predicting the clinical efficacy of OVs remain lacking, and related research is still in its exploratory phase. Future efforts should integrate multidimensional data (encompassing TME characteristics, systemic immune status, and whole-body responses) and establish standardized evaluation systems through large-scale clinical validation [21].

Against this backdrop, a central question emerges: how can mechanistic insights into OV biology be systematically translated into rational design, delivery, and clinical application strategies? In this review, we aim to bridge fundamental mechanisms with clinical development by critically examining how viral engineering, delivery platforms, and combination regimens can be optimized to overcome current bottlenecks in efficacy, safety, and durable clinical responses. Rather than providing a descriptive summary, we focus on an integrative framework that links mechanistic principles to translational decision-making in oncolytic virotherapy.

To summarize, OVs have provided novel strategies for tumor therapy, positioning them as a promising component of the next generation of immunotherapeutic drugs. The rapid development of genetic engineering has made OV modification a research hotspot, enhancing their antitumor efficacy through exploration of new virus types, targets, administration routes, and combination therapies.

Looking forward, the key to future research lies in comprehensively considering the balance among viral characteristics, TME features, and host immune status. Efforts must focus on the diversification and optimal design of viral vectors. Ultimately, it is essential to develop a new generation of OV platforms that combine persistent infection capability, controllable pro-inflammatory activity, efficient induction of antitumor immunity, and favorable safety profiles.

### Classification of OVs and their clinical applications

A diverse range of viruses have been developed as OVs, including adenovirus (ADV), herpes simplex virus (HSV), measles virus (MV), Newcastle disease virus (NDV), reovirus (Reo), vesicular stomatitis virus (VSV), and coxsackievirus. ADV and HSV-1 are the most extensively investigated due to their well-characterized genetic modification techniques [29].

**Fig. 1:**
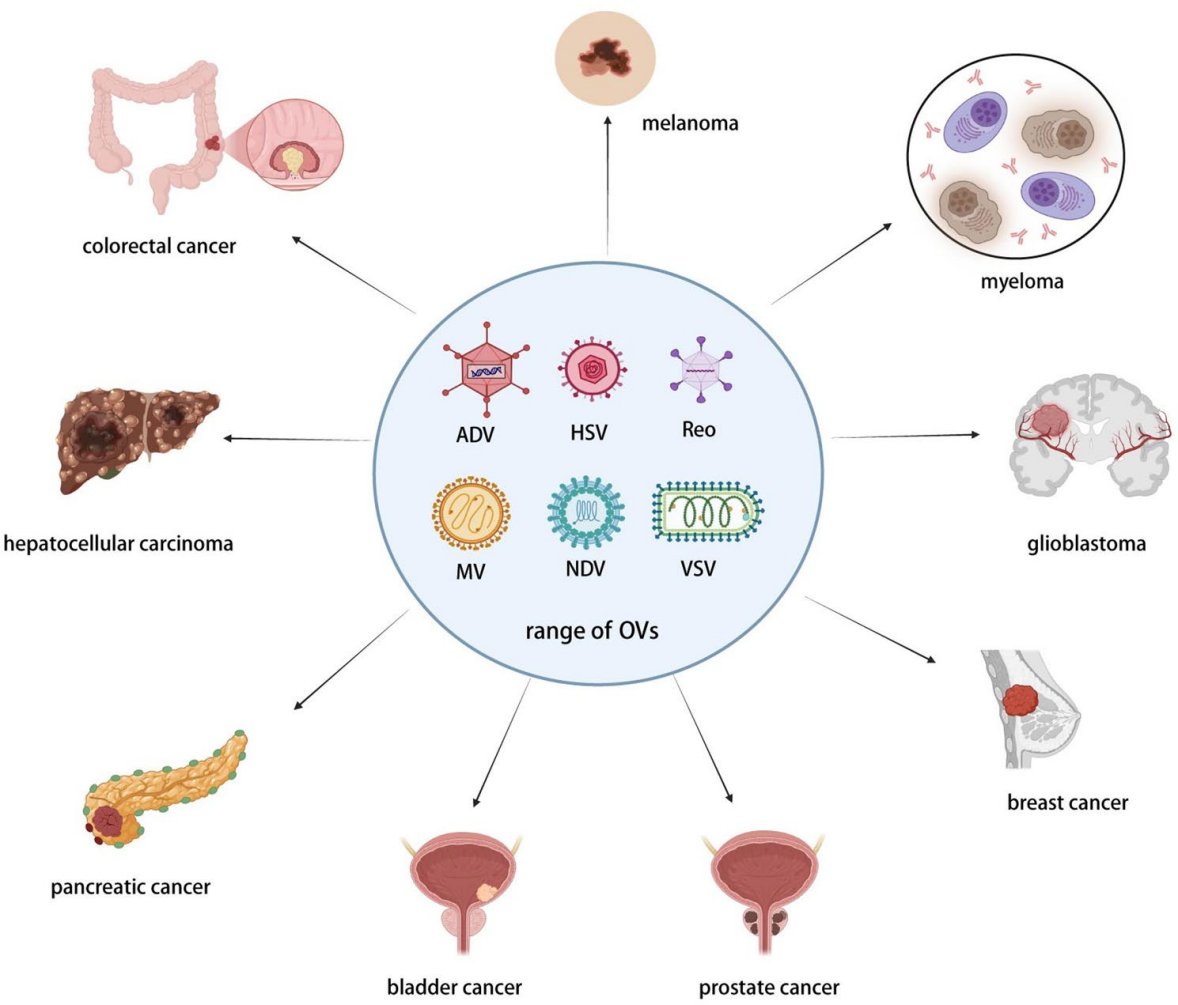
Clinical landscape of oncolytic viruses. A diverse range of viruses have been developed as OVs, and oncolytic virotherapy has now advanced to clinical applications across multiple cancers. ADV: Adenovirus, HSV: Herpes simplex virus, Reo: reovirus, MV: measles virus, NDV: Newcastle disease virus, VSV: vesicular stomatitis virus. Created with BioRender.com

Oncolytic virotherapy has progressed to clinical application across multiple malignancies, demonstrating therapeutic potential in brain tumors, melanoma, ovarian, endometrial, lung, and pancreatic cancers. Over 200 OV-related clinical trials have been conducted globally [25, 29] (Fig. 1).

Collectively, more than 200 clinical trials across diverse viral platforms and tumor types have established the overall safety and feasibility of oncolytic virotherapy. Treatment-related adverse events are predominantly low-grade and self-limiting, most commonly presenting as flu-like symptoms, regardless of viral backbone or route of administration [30]. While objective response rates observed with OV monotherapy are generally modest, often below 20%, clinical benefit frequently correlates with biomarkers of immune activation, including increased tumor-infiltrating lymphocytes and systemic cytokine responses.

These observations reinforce the concept that OVs function primarily as immune-priming agents rather than direct cytotoxic drugs. Accordingly, clinical efficacy appears to depend less on the intrinsic lytic capacity of individual viruses and more on their ability to consistently initiate and sustain antitumor immune cascades.

### Adenoviruses

ADVs are the most widely used and deeply studied OV platform. rAd.mDCN.mCD40L (driven by TERTp) effectively induces cytotoxicity in colorectal cancer (CRC) cells, inhibits metastasis, and remodels the TME by promoting Th1 immunity. Ad[CE1A] (driven by the SLAMF7 promoter) targets myeloma cells, enhances ICD, and shows synergistic effects when combined with clinical anti-myeloma drugs [31, 32]. Furthermore, recent studies engineered ADVs targeting distinct immune checkpoints to overcome resistance to immune checkpoint inhibitors (ICIs) [33–35].

ADVs are also extensively investigated in pancreatic cancer (PDAC): VCN-01 expresses hyaluronidase to degrade tumor stroma, enhancing drug delivery and showing efficacy in combination with chemotherapy (NCT02045589) [36]. LOAd703, encoding TMZ-CD40L and 4-1BBL, activates cytotoxic T cells and is being evaluated with chemotherapy for advanced PDAC [37].

### Herpes simplex virus

HSV-1-based OVs are prominent. T-VEC, the first OV approved by the US FDA (2015), is derived from HSV-1 and modified to express GM-CSF, enhancing immune activation [38]. T-VEC achieved significant clinical success in advanced melanoma. In brain tumors, CAN-3110 showed tumor-selective replication and good safety in recurrent glioblastoma (rGBM) patients [39]. VG161, an engineered HSV-1 co-expressing multiple immune modulators, showed favorable efficacy in hepatocellular carcinoma (HCC) [23]. In glioblastoma(GBM), G47Δ, a third-generation HSV-1, demonstrated a survival benefit and favorable safety in rGBM, leading to its approval in Japan [40, 41]. Additionally, the combination of orienX010 with PD-1 inhibitor toripalimab demonstrated efficacy in acral melanoma (AM) [42, 43].

### Other OVS

Heterologous oncolytic VSV-based strategies (e.g., VSV-IL-2) significantly enhanced the function and persistence of antigen-specific CD8 + T cells, demonstrating superior tumor suppression in melanoma models [44].

Coxsackievirus A21 (CVA21) and MV utilize specific receptors (ICAM-1 and CD46, respectively) highly expressed on breast cancer cells for tumor-selective infection [45–47].

Vaccinia virus (VV), a DNA virus with a large cloning capacity and robust lytic potential, has been extensively engineered to enhance antitumor immunity [48–51].

Furthermore, OVs also show potential for cancers with limited options: HSV, Reo, MV, PV, and ADV are in clinical trials for malignant brain tumors [46, 52]. OVs have confirmed therapeutic potential against triple-negative breast cancer (TNBC) [46, 53]. Likewise, combining OVs with ICIs has shown synergistic antitumor effects in multiple preclinical and clinical bladder cancer models [54], and related clinical trials are advancing for both non-muscle-invasive and muscle-invasive subtypes [55].

Building upon the substantial progress achieved in recent years, it is evident that oncolytic virotherapy has emerged as a promising therapeutic modality for multiple solid tumors. However, despite these encouraging developments, research focusing on gastrointestinal (GI) malignancies—such as gastric, colorectal, and esophageal cancers—remains relatively limited compared with other cancer types. Notably, GI cancers account for over 25% of all cancer cases worldwide and nearly one-third of cancer-related deaths. Epidemiological data indicate that East Asia bears the heaviest burden of these cancers, with a notable rise in early-onset CRC incidence in recent years [56]. Given this substantial disease burden and the unmet therapeutic needs, there is an urgent need to intensify innovative research on OVs as a next-generation immunotherapeutic approach, aiming to better integrate them into comprehensive treatment strategies for GI malignancies [57].

Current clinical trials investigating OV-based therapies face several major limitations. Current OV clinical trials face major limitations: first, small sample sizes restrict statistical power [35, 43, 58, 59]. Second, systemic administration often results in insufficient tumor targeting [60]. Third, there is a lack of predictive biomarkers and a challenge in streamlining clinical translation [61–63].

To overcome these, future studies must expand clinical sample sizes, optimize administration routes for enhanced tumor-specific delivery, and develop reliable biomarkers for efficacy prediction. Tailored viral constructs and combination strategies are essential, especially given the distinct tumor microenvironment(TME) of GI cancers. Standardization of dosage, delivery route, and treatment timing is also critical. Supplementary information on the various types of OVs used in clinical trials and their corresponding target cancers is summarized in Table 1. (Table 1).

**Table 1:** Types of oncolytic viruses (OVs) used in clinical trials and their respective targets

virus	Combination therapy	Target	key mechanistic insight or outcome	Phase	Trial ID/Reference
**adenovirus**					
**NG-350 A**	-	metastatic/advanced epithelial tumors	dose-dependent pattern	I	ClinicialTrials.gov: NCT03852511
	pembrolizumab	metastatic/advanced epithelial tumors	Intravenous dosing	I	ClinicialTrials.gov: NCT05165433
	chemoradiotherapy	metastatic/advanced epithelial tumors	Intravenous dosing	I	ClinicialTrials.gov: NCT06459869
**Ad5-yCD/mutTKSR39rep-hIL-12**	-	recurrent prostate cancer (T1c-T2)	Topical administration, well-tolerated	I	ClinicialTrials.gov: NCT02555397
**TILT-123**	-	Advanced solid tumors	local and distant Antitumor activity, well-tolerated	I	ClinicialTrials.gov: NCT04695327
**VCN-01**	standard chemotherapy	Pancreatic ductal adenocarcinoma (PDAC)	stromal disruption capability	I	ClinicialTrials.gov: NCT02045589
**rAd.mDCN.mCD40L**	-	Colorectal cancer (CRC)	Immunomodulation, tumor microenvironment remodeling	Preclinical	15
**Ad[CE1A]**	bortezomib melphalan panobinostat pomalidomide	multiple myeloma	Highly potent and selective cytotoxicity	Preclinical	16
**LOAd703**	chemotherapy	advanced pancreatic ductal adenocarcinoma	Some treatment-related adverse effects	I/II	ClinicialTrials.gov: NCT02705196
**DNX-2401**	pembrolizumab	recurrent glioblastoma	safety endpoint met, efficacy endpoint not met.	I/II	ClinicialTrials.gov: NCT02798406
**H101**	-	Malignant ascites	Immunomodulation, well-tolerated	II	ClinicialTrials.gov: NCT04771676
**herpes simplex virus 1**					
**CAN-3110**	-	recurrent glioblastoma (rGBM)	Significant association between seropositivity and survival	I	ClinicialTrials.gov: NCT03152318
**G47∆**	Radiation temozolomide	residual or recurrent glioblastoma	Increased intratumoral immune infiltration, safe	II	UMIN: UMIN000015995
	Radiation temozolomide	residual or recurrent glioblastoma	Increased intratumoral immune infiltration, safe	I, II	UMIN: UMIN000002661
**orienx010 (ori)**	anti-PD-1 toripalimab (tori)	acral melanoma (AM)	Grade 1–2 adverse events, Elevated inflammatory cytokines	I	ClinicialTrials.gov: NCT04197882
**T-VEC**	pembrolizumab	advanced melanoma	No significant improvement in safety or efficacy	III	ClinicialTrials.gov: NCT02263508
	pembrolizumab	liver metastases hepatocellular carcinoma	Limited efficacy across tumor types	Ib/II	ClinicialTrials.gov: NCT02509507
	pembrolizumab	Locally Advanced or Metastatic Sarcoma	antitumor activity, manageable safety	II	ClinicialTrials.gov: NCT03069378
**T-VEC**	pembrolizumab	PD-1-refractory melanoma	antitumor activity, well-tolerated	II	ClinicialTrials.gov: NCT04068181
	BDCA-1 myDC BDCA-3 myDC (BDCA+Myeloid Dendritic Cells)	immune checkpoint inhibitor-refractory melanoma	Feasible, well-tolerated, Intratumoral administration	I	ClinicialTrials.gov: NCT03747744
	nivolumab ipilimumab	localized HER2-negative breast cancer	antitumor activity long-term toxicity	I	ClinicialTrials.gov: NCT04185311
**coxsackie virus**					
**A21(V937)**	pembrolizumab	advanced solid tumors	Manageable intravenous safety, efficacy not improved	I	ClinicialTrials.gov: NCT02043665
	pembrolizumab	resectable stage IIIB-D melanoma	manageable safety	I/II	ClinicialTrials.gov: NCT04303169
	pembrolizumab	advanced melanoma	Robust treatment response, manageable safety	Ib	ClinicialTrials.gov: NCT02307149
	pembrolizumab	metastatic/unresectable stage melanoma	Overcomes microenvironmental limitations	Ib	ClinicialTrials.gov: NCT02565992
**newcastle disease virus**					
**MEDI5395 (NDV-GM-CSF)**	durvalumab	advanced solid tumors	a trend toward dose-dependent pharmacokinetics	I	ClinicialTrials.gov: NCT03889275
**vaccinia virus**					
**JX-594**	-	refractory primary or metastatic liver cancer	antitumor activity manageable safety	I	ClinicialTrials.gov: NCT00629759
	Chemotherapy Avelumab	Advanced Breast Cancer Advanced Soft-tissue Sarcoma	Data not yet available	I/II	ClinicialTrials.gov: NCT02630368
**measles virus**					
**MV-NIS**	-	Pediatric recurrent medulloblastoma, atypical teratoid/rhabdoid tumor (ATRT)	Data not yet available	I	ClinicialTrials.gov: NCT02962167
	-	multiple myeloma	maximum tolerated dose (MTD) established	I	ClinicialTrials.gov: NCT00450814
**Parvovirus(H-1PV)**					
**ParvOryx01**	**-**	rGBM	Induces antibody production and antigen-specific T-cell responses	I/IIa	ClinicialTrials.gov: NCT01301430
**vesicular stomatitis virus (VSV)**					
**VSV-IFNβ-NIS**	**-**	T-cell lymphoma (TCL)	Manageable single intravenous safety, dose-dependent efficacy	I	ClinicialTrials.gov: NCT03017820
**Poliovirus**					
**PVSRIPO**	**-**	grade IV malignant glioma	Intratumoral infusion no neurotoxicity	I	ClinicialTrials.gov: NCT01491893

trials utilizing the following ovs are presented : hsv-1, adenoviruses, measlesviruses, vaccinia, coxsackie virus, newcastle disease virus, etc

bold text indicates the oncolytic virus types and their names

inclusion criteria: Viruses selected based on (i) clinical phase (I–III), (ii) genetic engineering for enhanced efficacy/selectivity, (iii) diverse administration routes, (iv) combination therapy data, and (v) reported mechanistic/immunological outcomes

### Anti-tumor activity of OVs: mechanisms

**Fig. 2:**
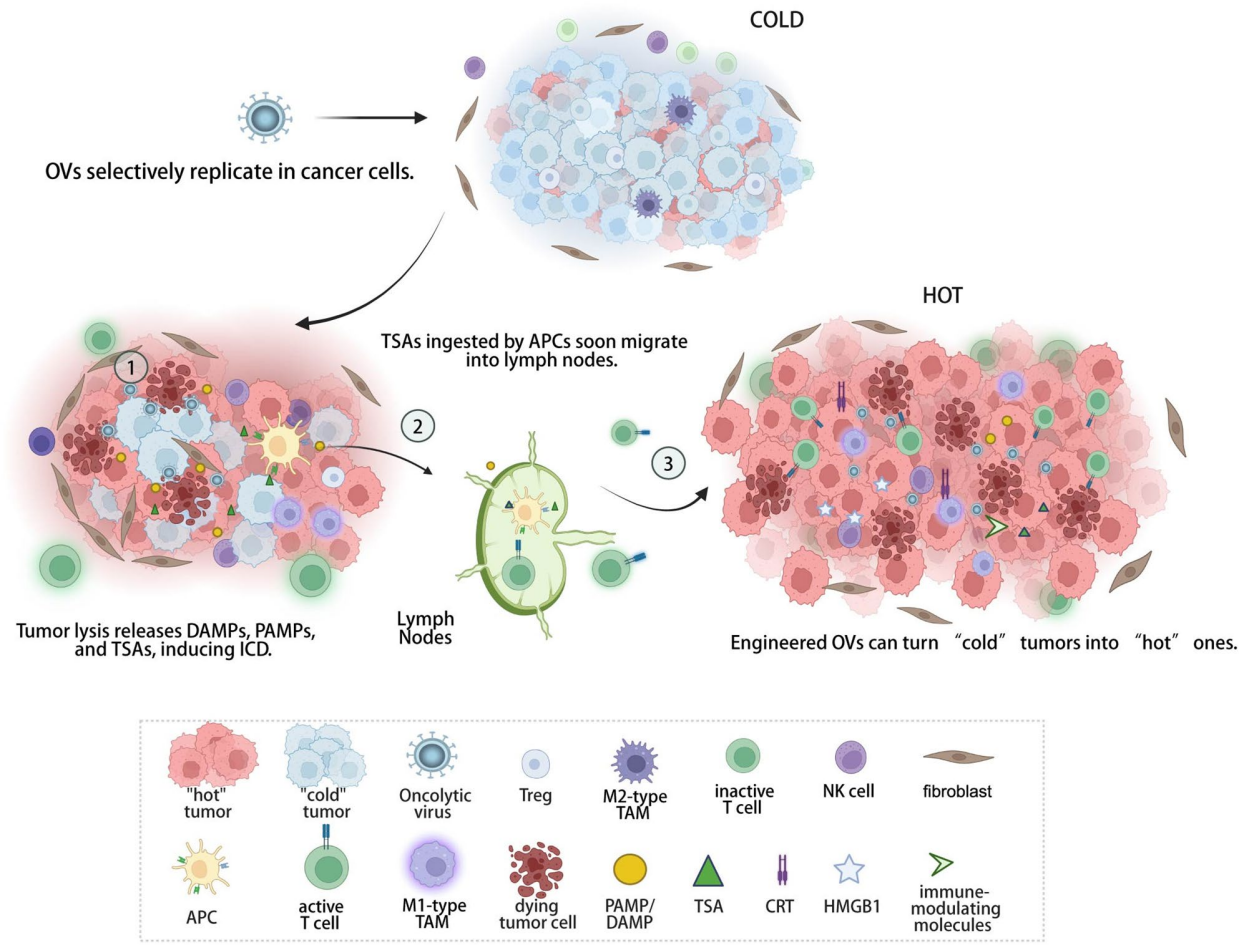
OV-mediated oncolysis–immune activation cascade. The anti-tumor activity of OVs does not rely solely on direct cell lysis but is achieved through a cascade reaction of " oncolysis-immune activation.” ①OVs specifically infect and replicate within susceptible cancer cells, and successfully cause direct oncolysis. Direct lysis of tumor cells releases DAMPs, PAMPs, as well as TSAs inducing ICD.②TSAs ingested by APCs soon migrate into lymph nodes, where T cells are activated, which infiltrate primary and metastatic site to perform adaptive immunity. ③The ICD pathway involves, after OV infection, the exposure of CRT on the tumor cell surface (“eat me” signal), release of HMGB1 (recruiting DCs), and secretion of ATP, forming a “danger signal cluster. In addition, engineered OVs by expressing immune-modulating molecules and editing TME components (e.g. suppressing Tregs, polarizing M1 macrophages), they break immune suppression. In a collaborative effort, the engineered OVs may transform the immunologically “cold” tumor into “hot” tumor, also exerting an upgraded and more powerful antitumor immunity. OV: oncolytic virus, DAMPs: Damage-associated molecular patterns, PAMPs: Pathogen-Associated Molecular Patterns, TSA: tumor specific antigen, ICD: Immunogenic Cell Death, APC: Antigen-Presenting Cell, CRT: calreticulin, HMGB1: high mobility group box 1, DCs : dendritic cells, ATP: activating purinergic receptors, TME: tumor microenvironment. Created with BioRender.com

The anti-tumor activity of OVs does not rely solely on direct cell lysis but is achieved through a cascade reaction of " oncolysis-immune activation.” Its core mechanisms can be divided into selective replication-mediated cell killing and systemic immune response activation through danger signal release and TME remodeling [21, 62, 64, 65] (Fig. 2).

### Direct oncolysis

Direct oncolysis constitutes the foundation of OV therapy. These viruses selectively infect and replicate in cancer cells, a process influenced by viral type, dose, tropism (inherent or genetically engineered targeting capability), and the intrinsic sensitivity of tumor cells to death pathways (apoptosis, necrosis, pyroptosis, autophagy) [66, 67]. Upon infection, OVs replicate and lyse tumor cells, releasing progeny virions that spread to adjacent cells, forming a self-amplifying lytic cycle.

Different types or engineered variants of viruses employ distinct mechanisms to achieve selectivity. Among DNA viruses: HSV-1 deletes the neurovirulence gene γ34.5(avoiding damage to normal cells) and leveraging active DNA replication in tumor cells (e.g., ICP6-deficient strains only replicate in S-phase cells) [68, 69]; Engineering vaccinia virus (VV) to express a target gene for specific small molecules under the control of an inducible promoter enables chemogenetic regulation of viral replication and transgene expression with spatiotemporal precision for tumor-selective targeting [70]. Complementarily, the replication deficiency of TK(thymidine kinase)- and RR(ribonucleotide reductase)-deleted VV in normal cells, resulting from disrupted nucleotide metabolism, restricts productive replication to tumor cells [48]. Among RNA viruses: The oncolytic selectivity of both MV and VSV relies on impaired interferon (IFN) pathways in tumor cells [71–74]; Coxsackievirus A21 (CVA21) targets ICAM-1/DAF receptors overexpressed on tumor cells [75]. However, selective replication of OVs faces two major limitations: first, some tumors may have defective IFN pathways but their dense collagen matrix hinders viral spread, leading to “efficient replication but inefficient diffusion” [63, 76–78]; second, some viruses have small genome capacities, making it difficult to carry both selective replication elements and therapeutic transgenes simultaneously. Future strategies may combine TME-modifying agents or dual-promoter systems to enhance selectivity.

OVs also induce systemic antitumor immunity, including abscopal effects and immune memory. An oHSV–GM-CSF platform suppressed both injected and contralateral metastatic lesions in MBM [79] ; EGFP-oHSV1–treated glioblastoma mice achieved 45% long-term survival and rejected rechallenge [68]; OncoVEXmGM-CSF with anti-CTLA-4/PD-1 achieved complete rejection of B16F10 rechallenge but not unrelated tumors, confirming tumor-specific memory [32, 80].

### Indirect immunomodulation: danger signal release and TME remodeling

The indirect anti-tumor immune mechanism plays a crucial role in amplifying and sustaining the therapeutic efficacy of OVs. The traditional view held that OV immune activation relied on the “inflammatory response triggered by viral infection, However, recent studies indicate that OVs activate immunity through two non-mutually exclusive pathways: first, direct infection and lysis of tumor cells by OVs simultaneously release Pathogen-Associated Molecular Patterns (PAMPs) derived from the virus and Damage-associated molecular patterns (DAMPs)( e.g. calreticulin (CRT), high mobility group box 1 (HMGB1), ATP), inducing immunogenic cell death (ICD); second, by expressing immune-modulating molecules (e.g., GM-CSF, IL-12) or editing TME components (e.g., suppressing Tregs, polarizing M1 macrophages), they break immune suppression [48, 66, 74, 81–85]. These molecules function as potent danger signals and inflammatory mediators, initiating a robust local inflammatory response. Following OV infection, ICD is characterized by CRT exposure on the tumor cell surface as an “eat me” signal, release of HMGB1 to recruit DCs, and secretion of ATP to activate purinergic receptors, collectively forming a coordinated danger signal cascade. For instance, following its administration to glioma-bearing mice, oHSV induces the release of HMGB1, which subsequently modulates the production of pro-inflammatory cytokines and inflammation [84]; The oncolytic NDV elicits the exposure of CRT and the release of HMGB1 and HSP70/90, along with ATP secretion in melanoma cells, thereby inducing ICD [86]; NDV activated cGAS/STING and enhanced NK cytotoxicity via type I IFN induction [87, 88]. These cascades recruit and activate immune cells while reducing immunosuppressive populations (e.g., regulatory T cells (Tregs), M2-type tumor-associated macrophages (TAMs)), converting “cold” TMEs into “hot” immunogenic milieus [64].

More profoundly, OV infection can induce a systemic anti-tumor immune response. OV-induced lysis releases tumor-associated antigens (TAAs) and neoantigens, which are captured by DCs and presented to activate tumor-specific CD8⁺ CTLs, driving targeted cytotoxicity and systemic antitumor immunity. Some OVs also disrupt tumor vasculature or serve as transgene delivery vectors. Furthermore, certain OVs have been shown to disrupt tumor neovasculature, impacting tumor nutrient supply and metastatic potential. Additionally, OVs can impede tumor progression by disrupting tumor vasculature and/or serving as gene delivery platforms to enable the expression of therapeutic transgenes with anticancer effects [64, 89–92].

Adaptive immune remodeling is evident across models. An oHSV engineered to express a Claudin18.2/CD3 bispecific T-cell engager (OV-BiTE) enhances the infiltration of CD4 + T cells into the TME, reduces Tregs, and leads to TME remodeling [82]; IL-12-expressing Ad (Ad-siERCC1) activated CTLs via IL-12/IFN-γ signaling, increasing granzyme B expression 2.3-fold [93]; VG161 (HSV-1–derived) expressing IL-12, IL-15/IL-15RA, and a PD-L1-blocking peptide raised intratumoral CD8⁺ T cells from 5% to 28% while reducing Tregs from 12% to 3% [94]. Macrophage polarization also contributes: MEM-288 (Ad encoding CD40L and IFNβ) increased the M1/M2 ratio from 0.3 to 1.8 in high-grade serous ovarian cancer (HGSOC) [95]; TG6050 (VV encoding IL-12 and anti-CTLA-4) tripled M1 macrophage proportions in B16F10 melanoma [48]; An OV expressing SIRPα-Fc (oAd-SA) enhanced macrophage phagocytic activity by blocking the CD47-SIRPα “don’t eat me” signal, while downregulating M2 markers (e.g., CD206) [96].

Nevertheless, immune activation varies. Some OVs recruit DCs but expand myeloid-derived suppressor cells (MDSCs), maintaining immunosuppression [97]; Strong constitutive promoters (e.g., CMV) may cause systemic toxicity from excess GM-CSF, while tumor-specific promoters (E2F) mitigate this [28]; In “cold” tumors such as PDAC, poor T-cell infiltration hampers secondary immune activation even with ICD induction [63, 76–78]. Future efforts need to optimize immune activation efficiency through “precision arming” or “TME preconditioning.”

Collectively, these findings highlight that the immunological effects of OVs are not incidental consequences of viral infection, but rather modular and tunable processes. Each component of OV-induced immune activation—including ICD induction, cytokine and chemokine signaling, antigen presentation, and immune cell reprogramming within the TME—represents a rational target for genetic and structural engineering. Consequently, contemporary OV development increasingly focuses on deliberately arming viruses with immunomodulatory payloads, optimizing transgene expression control, and reshaping virus–host interactions to maximize therapeutic immunity while minimizing off-target inflammation. These mechanistic insights provide the conceptual framework for the engineering strategies discussed in the following section.

### Basis for selective replication

Selective targeting of tumor cells underlies OV safety and efficacy. This specificity arises from aberrant receptor expression (e.g. SLAM/CD150, CD46,ICAM-1) mediating viral entry [98–100]. Engineering viral surface proteins enhances tropism toward tumor-specific receptors such as EGFR, improving infection efficiency [101]. 

Another determinant is the defective antiviral signaling in tumor cells. In normal cells, viral PAMPs activate PRRs (e.g., TLRs, RIG-I), leading to IRF3/IRF7-mediated IFN-I production and PKR activation, which halts protein synthesis and induces apoptosis, blocking viral spread. In contrast, tumor cells often harbor defects in the IFN or PKR pathways due to mutations (e.g., p53 loss, Rb dysregulation) or epigenetic changes (e.g., hTERT activation), rendering them permissive to viral replication [102–105]. These deficiencies paralyze antiviral defenses, allowing OVs to replicate selectively in malignant cells while sparing normal tissues [38].

One major engineering strategy to achieve tumor-selective replication in adenoviruses is the deletion of viral genes that are essential for replication in normal cells but dispensable in cancer cells due to oncogenic pathway dysregulation. These conditional replication adenoviruses (CRAds) exploit defects in tumor suppressor and cell cycle control pathways to restore viral replication selectively in malignant cells.

A representative example is ONYX-15 (H101), which harbors a deletion of the E1B-55 kDa gene. In normal cells, E1B inactivates p53 to permit viral replication; thus, its deletion prevents efficient replication in p53-intact cells. However, since 50–75% of pancreatic cancers exhibit p53 loss or mutation, E1B-deleted viruses can replicate preferentially in tumor cells [106]. Similarly, deletion of E1A exploits dysregulation of the Rb–E2F axis, common in pancreatic cancer due to mutations in genes such as CDKN2A. In this context, viral replication becomes independent of E1A-mediated pRb binding. Additional deletions, such as E1B-19 kDa (an anti-apoptotic Bcl-2 homolog), further enhance tumor selectivity by taking advantage of the aberrant apoptotic environment in cancer cells [107]. Notably, combinatorial deletions (e.g., E1A and E1B-19 kDa) can significantly improve selectivity without compromising viral potency and may synergize with chemotherapy [108].

Together, these studies illustrate that rational gene deletions enable adenoviruses to achieve tumor selectivity while retaining therapeutic efficacy.

Beyond adenoviruses, similar deletion-based strategies have been applied to other oncolytic viruses to exploit tumor-specific signaling abnormalities.

HSV mutants exploit tumor metabolic and cell cycle alterations. The hrR3 virus lacks the UL39 gene encoding ICP6, a viral ribonucleotide reductase homolog. Because ribonucleotide reductase activity is frequently upregulated in pancreatic cancer cells, viral replication can proceed selectively in malignant tissues [109]. Combinatorial strategies further enhance HSV-based oncolysis. For example, ganciclovir activation via viral thymidine kinase augments cytotoxicity, while derivatives such as NV1020 and NV1066 demonstrate increased efficacy when combined with radiation or hyperthermia. Notably, hyperthermia promotes viral replication through induction of heat shock protein 72 (Hsp72), whose anti-apoptotic activity creates a permissive intracellular environment that facilitates HSV propagation, thereby amplifying tumor cell killing [109–111].

Similarly, reoviruses selectively replicate in tumors with activated Ras signaling. Given that KRAS mutations occur in approximately 85–90% of pancreatic cancers, reovirus replication is preferentially supported in malignant cells [112].

Synthetic biology–based gene circuits have further refined tumor-selective viral replication by integrating tumor-specific promoters and microRNA (miRNA) inputs. For example, Huang et al. engineered a sensory switch circuit in which the AFP promoter drives a Gal4VP16 activator and mutually inhibitory repressors responsive to miR-142 and miR-199a-3p, thereby enabling tumor-restricted E1A expression and adenoviral replication [113, 114]. Similarly, incorporation of miRNA target sequences into viral genomes enhances specificity and safety. Jia et al. inserted dual miR-34a target sites into both the 5′UTR and 3′UTR of coxsackievirus B3, achieving maintained oncolytic potency with reduced off-target toxicity [115]. In HSV platforms, tumor-associated miRNAs have also been exploited; insertion of miR-21–binding sites into the 3′UTR of a dominant-negative UL9 gene allows oncogenic miR-21, commonly upregulated in cancer cells, to relieve replication blockade and selectively restore HSV propagation in malignant tissues [116].

These examples collectively demonstrate that tumor-selective replication of oncolytic viruses can be achieved through multiple complementary mechanisms. By leveraging aberrant metabolic activity, stress-response adaptations such as Hsp72 induction, oncogenic Ras signaling, and synthetic promoter–miRNA regulatory circuits, engineered OVs are able to preferentially propagate within malignant cells while limiting replication in normal tissues. Such strategies highlight how intrinsic hallmarks of cancer—including signaling dysregulation, altered apoptosis control, and miRNA reprogramming—can be rationally exploited to enhance both the specificity and therapeutic index of oncolytic virotherapy.

### Current strategies for engineering innovative OVs

**Fig. 3:**
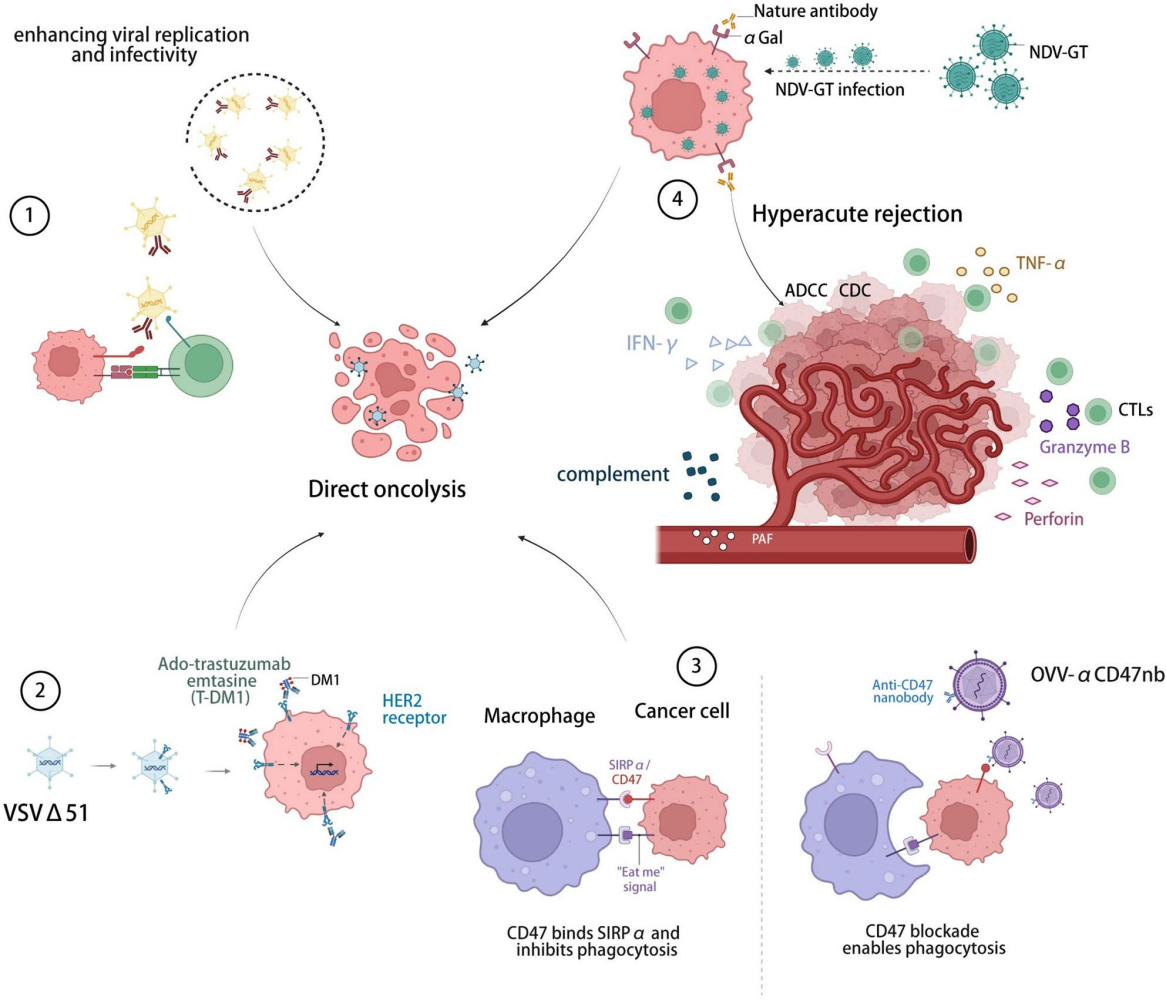
Genetic engineering strategies for optimizing oncolytic viruses. The modification of OVs is primarily achieved through genetic engineering approaches. (1) enhancing viral replication and infectivity specifically within tumor tissues: ① OVs expressing PD-L1 inhibitors block immune checkpoints and prolong viral replication in tumors. (2) improving tumor-targeting specificity and cytotoxicity: ②Engineered VSVΔ51 expressing HER2T enables HER2-targeted therapy, making tumors responsive to T-DM1.③OVV-αCD47nb blocks CD47–SIRPα signaling, activating macrophages to phagocytose tumor cells. (3) precisely modulating virus-induced immune responses: ④NDV-GT induces α-Gal–mediated hyperacute rejection, causing vascular damage and thrombosis that block tumor blood supply. OV: Oncolytic virus Created with BioRender.com

To overcome the inherent limitations of conventional OVs, one of the most effective approaches lies in their rational modification and optimization through genetic engineering. Currently, the modification of OVs is primarily achieved through genetic engineering approaches, with three key objectives: (1) enhancing viral replication and infectivity specifically within tumor tissues; (2) improving tumor-targeting specificity and cytotoxicity; and (3) precisely modulating virus-induced immune responses [21, 70, 117–119]. These engineered modifications allow OVs to evade premature clearance by the host immune system while maintaining low-level persistent replication in the TME, thereby achieving both therapeutic efficacy and favorable safety profiles [105, 106] (Fig. 3).

However, genetic engineering of OVs is not a simple additive process in which therapeutic functions are incrementally stacked onto a viral backbone. Instead, OV engineering requires navigating multiple intrinsic trade-offs, including balancing enhanced viral potency with safety constraints, immune evasion with immune activation, and transgene payload capacity with viral fitness and replicative competence. Each engineering strategy prioritizes different aspects of these competing demands, often improving one dimension at the expense of another. Understanding these trade-offs is therefore essential for critically evaluating current engineering approaches and for guiding the rational design of next-generation OV platforms.

### Enhancing viral replication and intratumoral persistence

OVs are often armed with cytotoxic agents, checkpoint inhibitors, modifiers of the innate immune response, and direct activators of T cells and NK cells [25, 120].Therefore, OVs can be engineered to express transgenes that inhibit the host’s anti-viral immune response, reducing viral clearance [121–123]. An OVs expressing a PD-L1 inhibitor (e.g., rPRV-iPD-L1) not only blocks the PD-1/PD-L2 immune checkpoint but also inhibits the activation of anti-viral T cells (PD-L1 is highly expressed on virus-infected cells), extending viral replication time in the tumor by 3 days [124]. An Ad expressing IL-10 (an immunosuppressive cytokine) can inhibit TNF-α release from macrophages, reducing viral clearance; in mice, viral titer increased 2-fold, but note that IL-10 may weaken anti-tumor immunity [68]. his strategy illustrates a fundamental trade-off: while suppression of antiviral immunity prolongs intratumoral viral persistence, excessive immunosuppression may concurrently blunt antitumor immune priming, thereby limiting durable therapeutic efficacy. Combining with immune suppressors can further inhibit anti-viral immunity. When TG6050 (encoding IL-12 and anti-CTLA-4) was combined with anti-PD-1, anti-PD-1 suppressed anti-viral CD8 + T cells while enhancing anti-tumor T cell activity, extending viral survival time in the tumor from 3 days to 7 days [48]; Cyclosporine A (inhibiting T cell activation) used in combination with Ad reduced anti-viral T cell infiltration, increasing viral replication efficiency 1.8-fold [70]. These studies highlight that enhancing viral persistence through immune suppression is highly context-dependent, requiring careful calibration to avoid undermining the immune-mediated antitumor effects that OVs are designed to elicit.

Synthetic biology enables the “customized construction” of OVs through “modular design” and “standardized parts”—for example, splitting the viral genome into multiple fragments for in vitro recombination assembly, or introducing “safety switches” to regulate viral replication and transgene expression, significantly improving the controllability and safety of OVs [69, 70].

Directed evolution involves serially passaging OVs in tumor cells to select for mutants with high affinity and high replication efficiency for specific tumors, enabling the rapid generation of “personalized OVs [125–127]. " Traditional directed evolution requires 10–50 passages (> 60 days), while novel methods (e.g., volume-dependent passage) can shorten the time to < 30 days [128]. Replication adaptive evolution can enhance viral replication efficiency and oncolytic activity. Coxsackievirus B3 (CVB3) PD-H strain was passaged in MC38 CRC cells using “volume-dependent passage” (instead of traditional dose-dependent); an adapted strain P-10 was obtained in just 10 passages, with a replication efficiency 10 times that of PD-H and oncolytic activity increased 3-fold, showing specificity for MC38 (no increased activity against other CRC cells) [129]; In refractory colorectal cancer HCT-116 cells, directed natural evolution of the M1 virus generated NGOVM, which exhibited a 9690-fold increase in oncolytic efficacy and a broadened antitumor spectrum. NGOVM maintained a favorable safety profile in both rodents and nonhuman primates [130]. VSV passaged in IFN-β-high-expressing PDAC cells yielded an IFN-resistant variant VSV-IFNres, whose replication efficiency in PANC-1 cells was 2.5 times that of wild-type VSV [127, 129, 130]. 

The screening process for these engineered OVs remains labor-intensive. However, a recent study has developed an efficient screening approach by leveraging the differential antibiotic susceptibility of viruses, combined with transposon-based insertional mutagenesis and nanopore sequencing technology. This methodology enables high-throughput identification of recombinant viruses capable of stable and safe expression of immunomodulatory factors, while simultaneously determining genes nonessential for viral replication in tumor cells. This breakthrough paves the way for accelerated engineering of OVs [131, 132]. Meanwhile, CRISPR/Cas9 modification enables efficient genome editing, targeted oncogene disruption, and restriction factor screening. CRISPR/Cas9 combined with the Sleeping Beauty (SB) transposon was used to construct mutant libraries for HSV-1 and VV, screening out HSV-3941 (UL39-UL41 deletion) and HSV-US24 (US2-US4 deletion); these mutants had better growth characteristics than the ICP34.5 deletion strain and were safe upon intracranial injection [131]; Using CRISPR/Cas9 to insert EGFP into the intergenic region between UL55-UL56 of HSV-1 created EGFP-oHSV1 with an editing efficiency of 70%, significantly higher than traditional homologous recombination (< 15%) [32, 133].

### Improving tumor targeting and cytotoxicity

Researchers have developed dual-targeted OVs to synergistically enhance tumor selectivity, therapeutic efficacy, and immune microenvironment remodeling [82, 134]. A major advantage of these multispecific agents is their ability to direct potent T-cell cytotoxicity against any engaged target cell, independent of T-cell receptor specificity or the human leukocyte antigen (HLA) status of the cancer cells [135]. By genetically engineering the oncolytic rhabdovirus VSVΔ51 to express a truncated target antigen (HER2T), HER2-targeted therapy was achieved using trastuzumab. Virus-infected tumors mimicked HER2-positive status, thereby responding to the antibody-drug conjugate T-DM1 in vitro, ex vivo, and in vivo. Furthermore, combining VSVΔ51-HER2T with an oncolytic VV expressing a HER2-targeted T-cell engager recruited CD3 + cell infiltration and established potent anti-tumor immunity, demonstrating strong curative effects. This study confirms the feasibility of remodeling the TME using OVs to improve compatibility with existing targeted therapies [136].

The scientific community is actively exploring novel strategies to engineer OVs based on transplant rejection mechanisms. Given their host-derived origin, cancer cells typically express self-MHC molecules that facilitate immune evasion [137, 138]. Importantly, as demonstrated in the aforementioned study, tumor progression necessitates escape from immune surveillance, whereas most current immunotherapies rely on MHC-restricted CD8 + T cell recognition of tumor antigens - a process that sharply contrasts with the hyperacute rejection in organ transplantation caused by MHC mismatch and preexisting antibodies. Building upon these findings, innovative approaches to genetically modify tumor cells to be recognized as “non-self” are providing new avenues for selective tumor elimination [139]. As referenced earlier, the recent groundbreaking study developed a recombinant oncolytic Newcastle disease virus expressing porcine α1,3-galactosyltransferase. This engineered virus demonstrated efficacy not only in a CRISPR-induced monkey hepatocellular carcinoma model but also achieved a 35% objective response rate in a phase I clinical trial involving 20 evaluable patients, primarily through inducing hyperacute tumor rejection [24].

### Precisely modulating virus-induced immune responses

Recent advances in engineered OVs targeting key signaling molecules have demonstrated remarkable potential for modulating tumors and their microenvironment [66, 140, 141]. In particular, OVs targeting the CD47-SIRPα axis have emerged as a research focus. CD47, a critical immune checkpoint molecule, delivers a potent “don’t eat me” signal by binding to SIRPα on phagocytic immune effector cells (e.g., macrophages). Studies show that blocking CD47-SIRPα interaction in the presence of pro-phagocytic signals potently activates TAMs to eliminate malignant cells [142].

Compared to systemic administration, intratumoral delivery of CD47-blocking OVs offers distinct advantages: targeted and sustained drug release within the TME while potentially minimizing systemic toxicity. A recent breakthrough involved an oncolytic VV encoding an anti-CD47 nanobody (OVV-αCD47nb), which locally disrupts CD47 signaling. This approach not only enhanced macrophage phagocytosis, suppressing breast and colon cancer progression and improving survival, but also reprogrammed the immunosuppressive TME—evidenced by increased T-cell infiltration, CD8 + T-cell activation, and TAM polarization. Combination with PD-1 blockade further improved efficacy, offering a novel translational strategy [49]. However, strategies that amplify immune activation enhance antitumor efficacy but often accelerate antiviral clearance, highlighting the need for precise control over the intensity and duration of immune stimulation.

The toxicity of OVs is a major limiting factor for dose escalation, making toxicity control a crucial research direction. Toxicity control modification reduces OV off-target toxicity and systemic inflammatory responses through gene deletion, promoter regulation, or transgene expression. Its core is the “precise regulation of viral activity or immune activation intensity,” finding a balance between efficacy and safety [143, 144]. Promoter selection and chemogenetic switches enable regulated transgene expression for toxicity control. Using medium-strength, tumor-specific promoters (e.g., E2F, survivin) to drive immune modulators (e.g., GM-CSF), instead of strong promoters (e.g., CMV), reduced systemic GM-CSF concentration by 90%, avoiding fatal CRS [28]; VV carrying a rapamycin-inducible ST7 system showed a 5-fold increase in transgene (e.g., GM-CSF) expression after rapamycin treatment, enabling temporal control [37].

Engineering OVs to express toxicity-neutralizing transgenes, such as cytokine antagonists and detoxifying enzymes, can reduce virus-mediated cell damage. An OV expressing IL-1 receptor antagonist (IL-1Ra) neutralizes IL-1β (an inflammatory cytokine), reducing toxicity like fever and weight loss; in mice, IL-1Ra expression reduced the rate of weight loss from 20% to 5%; Expressing a TNF-α antibody fragment neutralizes TNF-α-mediated hepatotoxicity, reducing ALT levels by 60% [145]; Expressing superoxide dismutase (SOD) clears ROS, reducing virus-mediated damage to normal cells [146]. These approaches primarily favor viral persistence and local immune modulation but must be carefully constrained to avoid excessive dampening of systemic antitumor immunity.

Nevertheless, immune escape modification needs to avoid “excessive suppression”—excessive shielding of viral immunogenicity may prevent OVs from activating anti-tumor immunity, and excessive suppression of anti-viral immunity may increase the risk of viral spread [66, 147]. The future requires the development of “spatiotemporal regulation systems” (e.g., expressing immunosuppressive molecules only after viral replication) to balance escape and immune activation. Therefore, future OV designs increasingly emphasize spatiotemporal regulatory systems, in which immunosuppressive or immunostimulatory modules are conditionally activated according to viral replication stage or tumor-specific cues. Such designs aim to reconcile viral persistence with effective antitumor immune activation, rather than optimizing either dimension in isolation.

The integration of genetic engineering and OVs represents a highly promising research direction for the future. In recent years, the integration of synthetic biology, CRISPR gene editing, directed evolution, and other technologies has further provided an efficient and precise tool for the modification of OVs, from “single gene deletion/insertion” to “multi-module precision design,” giving rise to a number of innovative modification methods that significantly enhance the customizability and efficacy of OVs.

### Delivery

Despite significant advancements in the mechanisms and therapeutic efficacy of oncolytic virotherapy, its clinical application continues to face major challenges in delivery strategies. Currently, the vast majority of FDA-approved anticancer drugs are administered systemically via intravenous or oral routes [148, 149], whereas oOVs remain primarily restricted to localized injection. However, this delivery approach has notable limitations: for tumors that have metastasized to distant sites or are located in deep-seated tissues, intratumoral injection fails to achieve comprehensive coverage, leading to residual tumor cells. Furthermore, the highly dense TME(e.g., the physical barrier posed by the extracellular matrix) further impedes efficient viral penetration and dissemination [147, 150]. Therefore, exploring more effective and versatile delivery strategies—such as systemic delivery, targeted delivery, or biomaterial-based sustained-release systems—is critical for advancing the clinical translation of oncolytic virotherapy.

Currently, various types of systemically deliverable viruses are under investigation. Among them, EnAd has been safely administered via intravenous injection due to its stability in whole human blood and the low pre-existing levels of neutralizing antibodies against adenovirus serotype 11 [151]; oncolytic VV and measles virus have demonstrated selective replication and transgene expression in tumor tissues following intravenous infusion [50]; Reovirus (Reo) has been routinely delivered intravenously, although its small size may limit its capacity to stably encode transgenes [135]. Notably, NDV-GT has also undergone intravenous administration in a clinical trial, demonstrating a favorable safety profile and supporting its potential as a systemically deliverable OV [24].

Based on the currently validated therapeutic targets, we can further expand our research approach by “arming” OVs to construct intelligent delivery systems that combine direct oncolytic effects with targeted therapeutic functions. For example, recent advances in synthetic virology have enabled the development of photocontrollable oncolytic mononegaviruses, in which Magnet proteins were inserted into the viral polymerase to allow blue light–dependent activation of viral replication. In these engineered measles and rabies virus platforms, viral replication and oncolytic activity were tightly regulated and occurred only upon light-induced polymerase dimerization, thereby providing spatiotemporal control over viral propagation. Such controllable OV systems represent a novel strategy to enhance therapeutic precision and minimize off-target toxicity while maintaining potent antitumor efficacy [152]. This innovative strategy not only achieves synergistic effects between oncolytic virotherapy and targeted therapy but also expands the therapeutic indications to more refractory tumor types by modulating the TME. For instance, an OX40L-armed oncolytic HSV-1 (OV-mOX40L) enhanced intratumoral CD4⁺ T cell activation, reduced regulatory T cell infiltration, reprogrammed myeloid cells toward a pro-inflammatory phenotype, and remodeled stromal components, thereby potentiating responses to immune checkpoint blockade.

Beyond immune costimulatory arming, antigen-redirection strategies have also been developed. An engineered OV encoding tumor-irrelevant bystander T cell (TBYS) epitopes (OV-BYTE) was designed to redirect pre-existing memory T cells toward infected tumor cells. By presenting viral or vaccine-derived epitopes within the tumor, OV-BYTE leveraged abundant, functionally preserved bystander T cells to mediate tumor killing and induced epitope spreading to broaden tumor-specific immunity. Notably, targeting SARS-CoV-2–specific memory T cells enabled effective tumor inhibition in preclinical models, illustrating how OVs can harness pre-existing antiviral immune memory to enhance antitumor efficacy [153, 154].

However, systemic delivery still faces significant challenges. A key concern is the persistence of OVs. The efficacy of OV therapy relies on their selective replication and spread within tumor tissues, enabling efficient tumor cell killing. However, this process is markedly suppressed by the host immune system’s rapid response [155]. OVs are easily cleared by neutralizing antibodies (NAbs), the complement system, and liver macrophages in the blood, resulting in less than 5% of the dose reaching the tumor site [128, 156, 157]. Following viral administration, the immune system recognizes PAMPs in the viruses, triggering innate immune activation and subsequent viral clearance [158, 159]. Consequently, the persistence of OVs may be relatively short-lived, thereby reducing therapeutic efficacy. Enhancing intratumoral OV persistence could thus be a critical strategy to improve treatment outcomes.

**Fig. 4:**
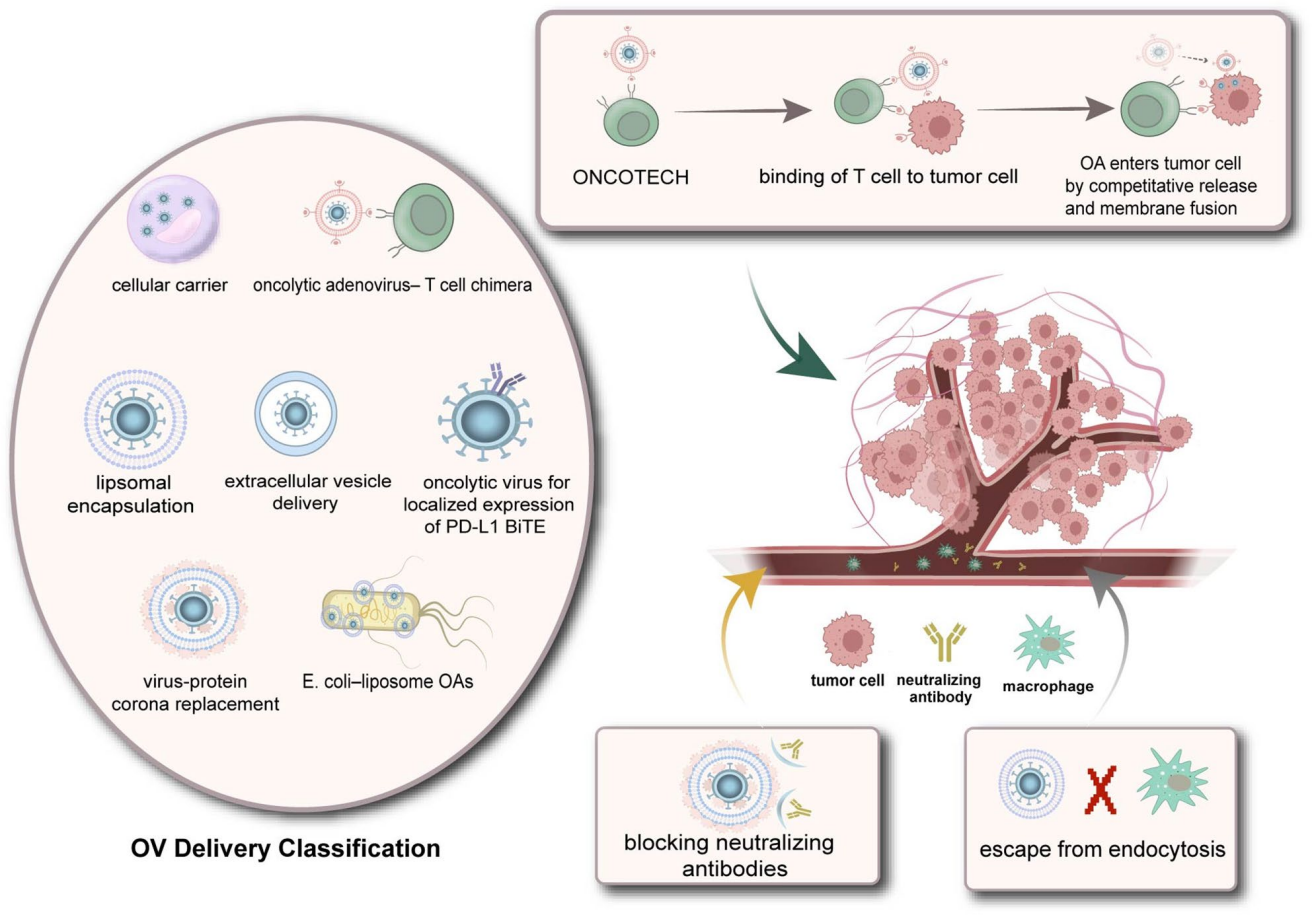
OVs delivery system optimization. Delivery system optimization, through strategies like carrier encapsulation, cell-mediated delivery and other innovative approaches can significantly enhance the in vivo stability, tumor targeting, and diffusion efficiency of OVs, representing a key solution to existing problems

Therefore, to maximize its oncolytic effects, effective viral antigen masking strategies must be explored to reduce host-mediated immune clearance, while a delivery system capable of promoting viral spread from the injection site to distant tumor masses and difficult-to-reach infiltrative cells would offer significant therapeutic advantages [160]. Delivery system optimization, through strategies like “carrier encapsulation,” “cell-mediated delivery,” or “physical assistance,” can significantly enhance the in vivo stability, tumor targeting, and diffusion efficiency of OVs, representing a key solution to these problems [161–163]” (Fig. 4).

### Using cell as a carrier

Cell-mediated delivery represents a promising strategy for OV therapy. By shielding the viral capsid from interactions with complement proteins and Factor X, cellular carriers can protect the virus from rapid clearance in the bloodstream. This approach not only facilitates transient immune evasion but also enhances tumor-specific targeting. Certain cell types, particularly stem cells, may further promote an immunosuppressive TME, thereby supporting viral replication and spread. As a result, cell-based vehicles can extend the therapeutic window for direct oncolysis by the virus, while the subsequent resolution of carrier-induced local immunosuppression may help initiate a potent antitumor immune response [164].

Cellular carriers include stem cell carriers and immune cell carriers, which can prolong the action time of the OVs and enhance anti-tumor immunity.

An engineered twin stem cell (TSC) platform carrying oHSV and GM-CSF: TSCs home to metastatic brain melanoma (MBM) via CXCR4 binding to SDF-1 in theTME. In an MBM model, the tumor homing efficiency of TSC-oHSV reached 80%, intratumoral virus quantity was 5 times that of free oHSV, and TSCs slowly released oHSV (for up to 7 days), preventing premature viral clearance; when combined with a PD-1 inhibitor, the inhibition rate of contralateral lung metastases reached 60%, significantly enhancing the abscopal effect [79].

Neural stem cells were the first cellular vehicle approved by the US Food and Drug Administration for the treatment of brain tumors. Currently, an ongoing clinical trial is evaluating the delivery of oncolytic adenoviruses using human mesenchymal stem cells in patients with recurrent malignant brain tumors [161]. Neural stem cell delivery of an oncolytic adenovirus was evaluated in a first-in-human, Phase 1, dose-escalation trial involving patients with newly diagnosed malignant glioma [165].

OVs not only enhance therapeutic efficacy when combined with CAR-T cells, but can also be efficiently delivered using CAR-T cells as carriers. Research has demonstrated that CAR-T cells can serve as effective vectors for systemic delivery of HSV to tumor sites. In a bilateral subcutaneous tumor model, intratumoral virus injection only led to reduction in the injected side, whereas intravenous infusion of HSV-loaded CAR-T cells significantly suppressed growth on both sides [162].Besides, a study developed a novel oncolytic adenovirus–T cell chimera (ONCOTECH) platform to address two major limitations of oncolytic adenoviruses (OAs): inefficient systemic delivery to tumors and their tendency to induce immune checkpoint expression. The approach involved engineering OAs to be conjugated onto T cells via antigen–receptor interactions using T cell–specific antigen-presenting membranes, thereby leveraging T cells for targeted tumor delivery [163].

The utilization of immune cells as carrier vehicles for OV delivery represents a promising strategy. The TME is characterized by extensive infiltration of diverse immune cell types. The accumulation of large numbers of immune cells has long been recognized as a hallmark feature of tumor biology, particularly in advanced disease stages and regions with high metastatic potential. Leveraging immune cell-mediated viral delivery capitalizes on these inherent conditions to enhance tumor eradication. Several immune cell types have been investigated for OV delivery, including MDSCs, T cells, cytokine-induced killer (CIK) cells, antigen-presenting cells, and cancer-associated fibroblasts [166].

In a metastatic breast cancer model, dendritic cells loaded with α-galactosylceramide effectively delivered oncolytic VSV-ΔM51 expressing fusogenic FAST proteins (VSV-p14 or VSV-p15) to tumor sites, activating NKT cells and enhancing anti-tumor immunity. When combined with systemic VSV-p14 or VSV-p15 administration, this DC-mediated NKT cell activation reduced lung metastases to undetectable levels in all treated mice and achieved 100% long-term survival, compared with only 40–70% survival in monotherapy groups. Surviving mice exhibited robust immune memory, with impaired tumor growth upon rechallenge and enhanced CD8 + T cell cytotoxicity and IFNγ production against tumor cells [167].

Macrophages carrying VSVΔM51 enter tumors via “efferocytosis” (phagocytosing tumor cell debris), while the macrophages polarize to the M1 type (pro-inflammatory, anti-tumor), further enhancing immune activation. In a lung cancer model, the tumor infiltration efficiency of macrophage-VSVΔM51 reached 70%, viral survival time in the tumor extended from 3 days to 7 days, intratumoral CD8 + T cell infiltration increased 4-fold, and the M1/M2 macrophage ratio increased from 0.3 to 1.8 ; When combined with anti-PD-1, mouse survival was extended by 60%, significantly higher than the monotherapy group [168, 169].

### Protective encapsulation

Nano-carriers (e.g., liposomes, polymers, protein nanoparticles) can protect OVs from clearance by NAbs and complement through physical encapsulation or chemical conjugation, while utilizing the enhanced permeability and retention (EPR) effect of tumors for passive targeting, or achieving active targeting through ligand modification.

Lipsomal encapsulation, a type of nano-carrier, represents a relatively well-established strategy for the delivery of OVs. Conventional cellular entry of viruses depends on the presence of specific receptors on the host cell membrane. In contrast, lipid nanoparticles can facilitate viral entry through membrane fusion or non-specific endocytosis, thereby circumventing this limitation [120].

Through strategic modification of liposomes, it is possible to not only protect OVs but also enhance their tumor-specific delivery and improve targeting efficiency. In one study, a novel pH-sensitive and EGFR-targeted liposomal system (designated as LP-GE-PS) was developed for the encapsulation of oncolytic adenovirus (oAd). By incorporating a pH-responsive polymer and an EGFR-targeting peptide, this system significantly improved the specificity and accumulation of oAd in EGFR-positive tumors, while reducing non-specific accumulation in the liver [128].

Polymer carriers, another type of nano-carrier, can protect the virus from clearance and enhance viral diffusion. To enhance therapeutic efficacy, VSVΔM51 was encapsulated within a branched polyethyleneimine (PEI)-hyaluronic acid (HA) complex. In this system, HA facilitates active targeting by binding to CD44 receptors overexpressed on tumor surfaces, while PEI shields the virus from complement-mediated clearance. In a pancreatic cancer model, the blood half-life of PEI-HA/VSVΔM51 was extended 3-fold compared to naked VSV, tumor viral load increased 4-fold, and HA could degrade the tumor ECM, extending the diffusion distance of VSVΔM51 from 100 μm to 300 μm ; When combined with hyaluronidase, tumor growth inhibition reached 80%, significantly higher than the naked VSV group (35%) [170].

Extracellular vesicles (EVs) facilitate intercellular signaling among various cell types within the TME, mediating drug resistance, metastasis, and immune responses. EVs represent a promising platform for drug delivery. EVs are derived from cells but are not cells themselves, endowing them with excellent biocompatibility [171]. By encapsulating and delivering OVs within these vesicles, host immune rejection against the viruses can be significantly reduced [172]. Furthermore, through engineering approaches, the targeting ability of EVs can be enhanced, thereby improving the precision of OV delivery. For instance, recent studies have developed PSMA-targeted, ROS-responsive extracellular vesicles to deliver oncolytic herpes simplex virus type 2 (OH2). This platform not only shielded the virus from systemic clearance but also achieved precise tumor localization and triggered local PD-L1 blockade, resulting in a 70% increase in tumor growth inhibition compared to free OH2 [173].

Nanotechnology-based drug delivery systems, which employ synthetic or biological nanoparticles to target anticancer agents to tumor sites, are being extensively investigated for a wide range of cancers [174, 175].

A study utilizing bioluminescence and fluorescence imaging techniques for in vivo tracking of EVs biodistribution clearly demonstrated the selective targeted delivery of particles to tumor tissues. Furthermore, by encapsulating drugs within EVs, the systemic effects of paclitaxel (PTX)-induced diffuse inflammatory responses were effectively mitigated. These findings strongly support the systemic administration of EV-based formulations, either alone or in combination with OVs or chemotherapeutic agents [176, 177].

Beyond extracellular vesicles, other strategies for the encapsulated delivery of OVs employ various biomaterials, including formulations like nanoparticles and polymeric particles [178], as well as approaches based on biomimetic mineralization [179].

### Physical assistance delivery: breaking physiological barriers and enhancing permeability

**Fig. 5:**
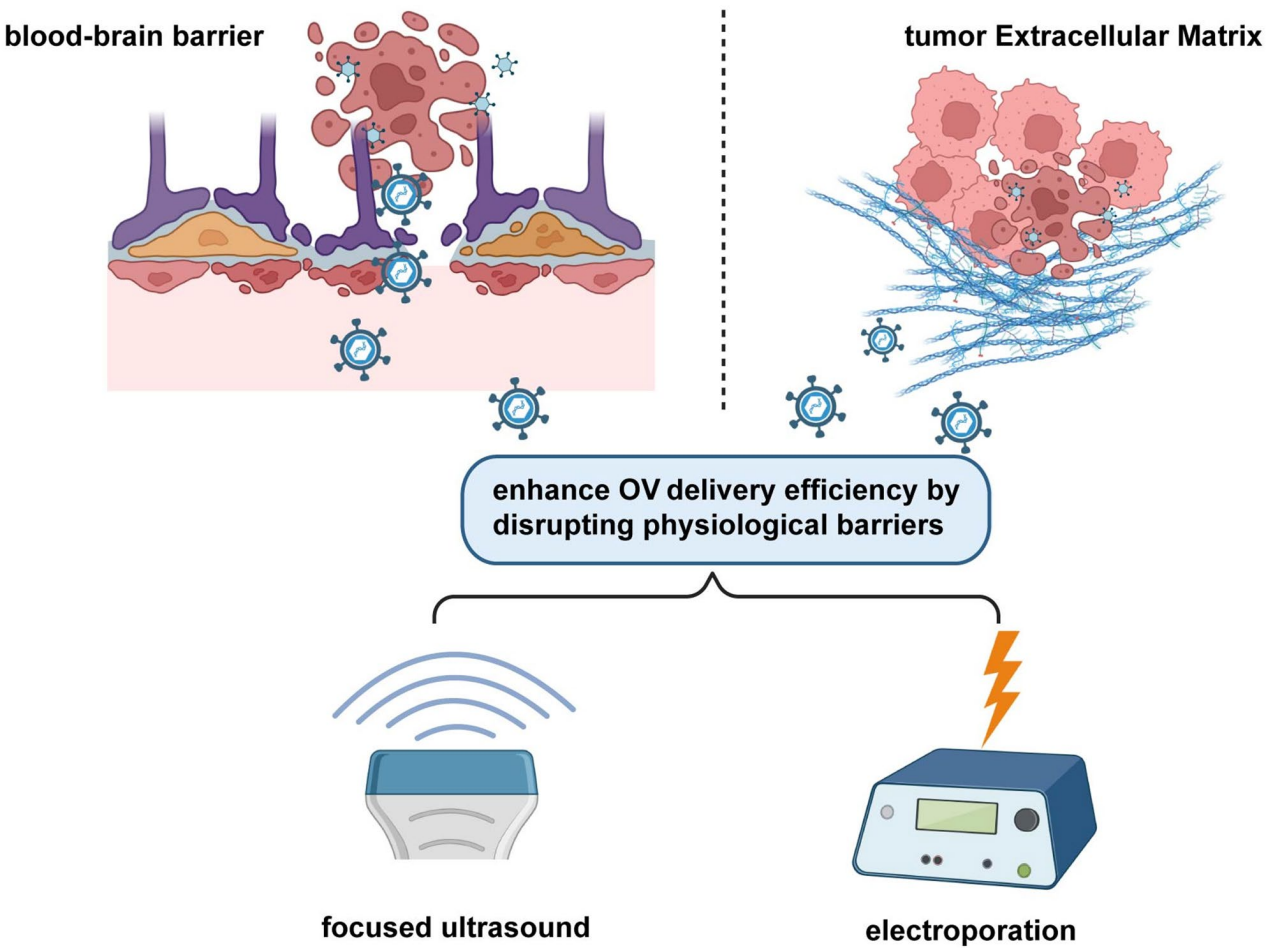
Physical methods enhance oncolytic virus delivery. FUS-MB transiently opens the BBB to boost CTV delivery in glioblastoma, while electroporation increases OncoVEX^mGM-CSF infection in melanoma and augments antitumor immunity. BBB: Blood-Brain Barrier, CTV: cancer terminator virus. Created with BioRender.com

Physical methods (e.g., focused ultrasound, electroporation, radiation) can enhance OV delivery efficiency by disrupting physiological barriers (e.g., blood-brain barrier (BBB), tumor ECM) or enhancing cell membrane permeability. Focused ultrasound-microbubble (FUS-MB) technology utilizes the mechanical effect of FUS to oscillate microbubbles, transiently opening the BBB (lasting ~ 4 h), while promoting OV entry into brain tumors. In a GBM model, with FUS-MB-assisted delivery of the cancer terminator virus (CTV), BBB permeability increased 3-fold, viral quantity in the brain tumor was 10 times that of intravenous administration, and CTV could selectively replicate in GBM via the PEG-Prom promoter (tumor-specific); tumor growth inhibition reached 70%, and mouse survival extended from 21 days to 45 days ; Multiple FUS-MB treatments (once every 7 days) could further improve efficacy, achieving a long-term survival rate of 35% [150]. Electroporation technology enhances cell membrane permeability through brief electrical pulses, promoting OV entry into tumor cells. In a skin melanoma model, intratumoral injection of OncoVEXmGM-CSF followed by electroporation increased cell membrane permeability 5-fold, achieving a viral infection efficiency of 80%, significantly higher than the injection-only group (30%); when combined with anti-CTLA-4, the complete tumor regression rate reached 40%, and immune memory was formed (100% rechallenge rejection rate) [80] (Fig. 5).

### Alternative innovative approaches

Recent studies are driving the optimization of OV delivery systems to enhance their therapeutic efficacy. For instance, a promising approach involves utilizing autonomously driven microbial vectors as systemic carriers. Researchers demonstrated this by combining liposome-encapsulated oncolytic adenoviruses (OAs) with tumor-homing E. coli BL21 (termed E. coli–liposome OAs), resulting in a striking enhancement of OA enrichment in non-small cell lung tumors by over 170-fold compared to naked OAs delivered intravenously [180].

In addition to genetic engineering, non-genetic surface modification provides a readily translatable alternative. One study proposed masking oncolytic HSV (oHSV) with a galactose-polyethylene glycol (PEG) polymer chain to minimize host antiviral responses and achieve selective tumor targeting by reducing systemic exposure following intravenous administration [181].

Moreover, improving the “defensive properties” of OVs is as crucial as boosting their “offensive capabilities.” Recognizing that rapid immune clearance is primarily driven by the formation of the virus-protein corona, a novel virus-protein corona replacement strategy was devised. This involves constructing an artificial protein corona on the OVs to shield them completely from key plasma components, shifting the research focus from inhibiting neutralization factors to modulating critical virus-protein corona interactions [182].

Crucially, the therapeutic potential of systemic delivery is underscored by Pexa-Vec (JX-594/TG6006), one of the most clinically investigated OVs. Intravenous administration of Pexa-Vec at a dose of 1 × 10⁹ plaque-forming units (p.f.u.) facilitates efficient viral delivery to tumor tissues, including micrometastases, and its association with plasma components supports tumor-specific viral trafficking and innate anti-cancer immunity [51].

Despite these significant advances, the optimization of OV delivery systems still faces several challenges. Specifically, carrier-virus interaction remains a hurdle, as some carriers (e.g., PEI) can non-specifically bind to the viral capsid, thereby compromising viral infectivity [86]. Similarly, safety concerns persist with cellular carriers, such as stem cells and macrophages, which may undergo abnormal proliferation or differentiation in vivo, often necessitating genetic editing tools like suicide genes to control their survival time [69]; (3) Lastly, the applicability of physical methods is limited; techniques like FUS-MB and electroporation require specialized equipment and are mainly suitable for superficial or accessible tumors (e.g., skin melanoma, brain tumors), with limited effect on deep-seated solid tumors (e.g., pancreatic cancer) [150, 170]. Therefore, the future necessitates the development of “intelligent delivery systems” or a sophisticated combination of multiple delivery strategies to further maximize OV targeting and efficiency.

### Strategic considerations for delivery platform selection

While diverse delivery strategies have been developed to improve the therapeutic index of OVs, no single platform is universally optimal. The choice of delivery method should be guided by the dominant biological barrier, tumor distribution, and translational feasibility. Here, we summarize key considerations to assist rational selection of OV delivery platforms across different clinical contexts (Table 2).

**Table 2:** Strategic considerations for delivery platform selection

Delivery Category	Key Challenges Addressed	Suitable Tumors/Scenarios	Advantages & Disadvantages	Clinical Translation Complexity
**Systemic Delivery**	Broad distribution to reach distant metastases	Disseminated disease; requiring systemic therapy	**Pros**: Wide coverage; good blood stability for some strains. **Cons**: <5% dose reaches tumor; rapid clearance; poor targeting.	Moderate
**Cell-mediated Delivery**	Immune evasion; Active targeting; TME modulation	Immune “cold” tumors; brain metastases; need for abscopal effects	**Pros**: Prolonged circulation; increased intratumoral load; synergistic immune activation. **Cons**: Safety risks (e.g., abnormal cell proliferation); complex manufacturing, high cost.	High
**Nanocarrier Encapsulation**	Immune shielding; Targeting ECM degradation; Improved stability	Solid tumors needing better targeting; deep-seated tumors; dense TME (e.g., pancreatic cancer)	**Pros**: “Stealth” prolongs half-life; smart responsive release; relatively mature platform. **Cons**: Carrier-virus interaction may reduce activity; challenges in large-scale production.	Moderate to High
**Physically Assisted Delivery**	BBB opening; ECM disruption; Enhanced local penetration	CNS tumors (e.g., GBM); superficial/accessible tumors (e.g., melanoma)	**Pros**: Direct, controllable barrier overcoming; immediate dose increase; combinable with other methods. **Cons**: Requires specialized equipment; limited to accessible sites; complex operation; difficult for deep/diffuse tumors.	Moderate
**Other Innovative Strategies**	protein corona modulation; bio-hybrid systems; surface chemistry	Seeking more effective immune escape; exploring new platforms; aiming for rapid translation	**Pros**: Protein corona offers new perspectives; microbial vectors show high enrichment; surface modification is simple. **Cons**: Mostly early-stage research; safety unverified; biosafety concerns for microbial vectors.	Moderate to High

*Abbreviations*: *BBB* Blood-brain barrier, *ECM* Extracellular matrix, *GBM* Glioblastoma, *TME* Tumor microenvironment

bold text indicates delivery categories, advantages and disadvantages

### Combination therapies with OVs

**Fig. 6:**
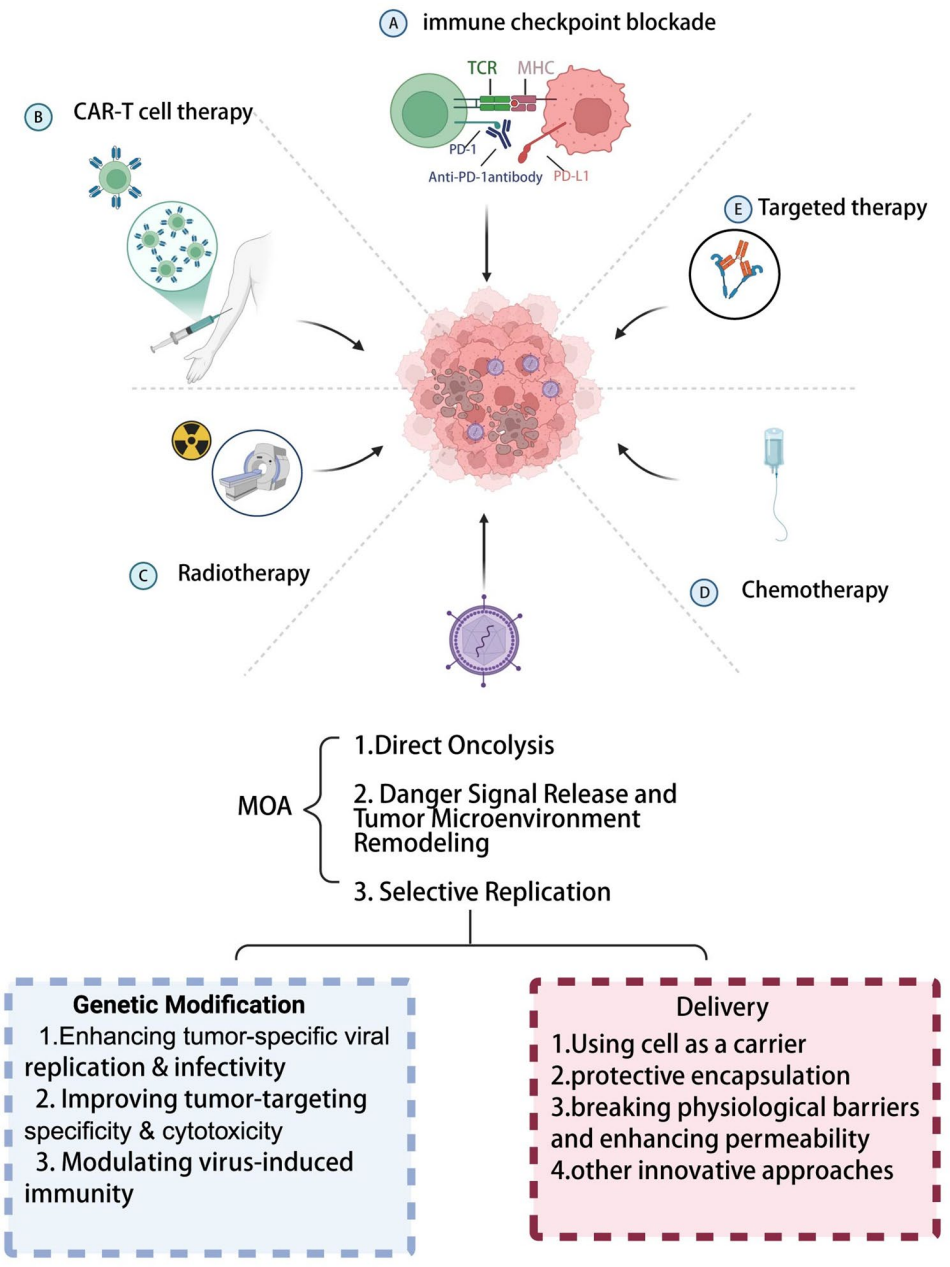
Synergistic combination strategies of oncolytic viruses with other anticancer therapies. Oncolytic viruses can enhance the efficacy of multiple therapeutic modalities by transforming “cold” tumors into “hot” tumors, improving immune cell infiltration, and modulating the tumor microenvironment, thereby achieving enhanced and durable antitumor responses. **A** Immune checkpoint blockade; **B** CAR-T cell therapy; **C** Radiotherapy; **D** Chemotherapy; **E** Targeted therapy. Created with BioRender.com

While OV monotherapy has demonstrated efficacy, its clinical application faces limitations. OVs exhibit distinct advantages in combination with conventional treatments (radiotherapy, chemotherapy, targeted therapy) and novel immunotherapies (CAR-T, ICIs ) [183–185] (Fig. 6). Beyond selecting combination partners, the sequence and timing of OV administration relative to the accompanying therapeutic modality may critically influence clinical outcomes. For example, administering OVs prior to immune checkpoint blockade may prime the TME through immunogenic cell death and T-cell recruitment, thereby enhancing responsiveness to ICIs. Conversely, OV delivery following CAR-T infusion may help eliminate antigen-negative escape variants or further remodel suppressive stromal barriers. Increasing preclinical evidence suggests that temporal coordination is an underexplored yet decisive variable in combination efficacy.

Immunotherapy with ICIs has been approved for the treatment of a wide range of cancers, including gastric cancer [186], metastatic melanoma, carcinomas of the head and neck, lung, kidney, and bladder, Merkel cell carcinoma, and Hodgkin disease [187]. However, ICI therapy does not elicit a response in all patients, largely due to the immunosuppressive TME [188]. OVs can convert “cold” tumors into “hot” tumors by reshaping the TME, making them particularly suitable for combination with ICIs. Rather than functioning as a merely additive strategy, OV–ICI combinations operate mechanistically: OVs initiate inflammatory priming through tumor cell lysis, antigen release, and local cytokine production, while ICIs sustain and amplify T-cell effector function [48, 189]. Combination therapies involving OVs and ICIs have advanced into clinical trials in numerous studies.

T-VEC, an OV, became the first widely approved OV product following its promising performance in clinical trials for metastatic melanoma. For instance, the combination of T-VEC with pembrolizumab has demonstrated preliminary safety and feasibility in treating HCC and liver metastases [190]. OVs are highly effective at overcoming the immunosuppressive TME) that limits ICI efficacy. This synergy is supported by robust clinical data. For instance, the combination of T-VEC with pembrolizumab has demonstrated preliminary safety and feasibility in treating HCC and liver metastases (102). Mechanistically, combination therapy utilizing T-VEC has been shown to enhance anti-PD-1 efficacy by modulating the TME, resulting in increased CD8 + T-cell infiltration, upregulation of PD-L1 expression, and enhanced IFN-γ gene expression [191].Furthermore, innovative engineering strategies directly address resistance; researchers successfully reversed TGFβ-mediated IFN-γsuppression using a TGFβRII inhibitor-armed VV, achieving systemic synergy [140].

Another team engineered three oncolytic adenoviruses (OAds) targeting specific immune checkpoints: OAd-SIRPα-Fc and OAd-Siglec10-Fc (macrophage-targeting) and OAd-TIGIT-Fc (T cell-targeting). These OAds modulated the TME and, when combined with anti-PD-1 (OAd-Siglec10-Fc), showed enhanced antitumor efficacy [33].Moreover, a VV expressing an anti-TIGIT antibody (VV-α-TIGIT) has been developed to induce long-term immunological memory. This effect relies on a CD8 + T cell-mediated mechanism to induce long-term immunological memory [192]. Clinically, the neoadjuvant combination of orienX010 with toripalimab showed promising antitumor activity and high response rates in resectable acral melanoma (AM) [42].

Beyond ICIs, OVs are critical for enhancing the efficacy of CAR-T cell therapy in solid tumors, where CAR-T activity is limited by the scarcity of tumor-specific target antigens, inefficient trafficking and infiltration of CAR-T cells into tumor sites, tumor heterogeneity with concomitant antigen escape, and a profoundly immunosuppressive TME [193].

OVs can enhance the efficacy of CAR-T cells through multiple mechanisms. For instance, genetic modification of OVs enables them to express cytokines, immune checkpoint regulators, or bispecific T cell engagers (BiTEs), which can improve the recruitment and infiltration of CAR-T cells into tumors. Additionally, such engineered viruses may boost the anti-tumor activity of CAR-T cells by modulating the TME or enhancing target antigen recognition, thereby significantly improving overall antitumor efficacy [194].

In a recent study, an oAds engineered to express the chemokine CXCL11 was developed to enhance CAR-T cell trafficking and reprogram the immunosuppressive TME [195]. Additionally, a study used a VV (OV19t) to induce *de novo* CD19 expression on tumor cells, thereby enabling effective CD19-CAR T cell targeting [196].

Importantly, the interaction between OVs and CAR-T cells is bidirectional. While OVs enhance CAR-T efficacy through microenvironmental remodeling and antigen modulation, CAR-T cells can also serve as active delivery vehicles for OVs, protecting them from systemic neutralization and facilitating targeted tumor deposition (see "Using cell as a carrier" section). For instance, HSV-loaded CAR-T cells achieved bilateral tumor suppression following intravenous infusion, whereas direct intratumoral viral injection produced only localized effects. Similarly, the ONCOTECH platform demonstrated that conjugating oncolytic adenoviruses onto T cells via antigen–receptor interactions enables efficient systemic tumor targeting while mitigating immune checkpoint upregulation. This reciprocal strategy transforms CAR-T cells from passive beneficiaries of viral priming into functional components of viral delivery systems, highlighting the integrated design potential of cellular–virotherapy platforms.

Additionally, researchers innovatively combined an OV (rVSV-LCMVG)—which is less prone to inducing neutralizing antibodies—with adoptive T-cell therapy in a study. By reprogramming the immunosuppressive TME, this approach significantly enhanced CD8⁺ T-cell infiltration and functional activation, resulting in synergistic antitumor effects. Furthermore, the research demonstrated that the combination of oncolytic virotherapy with an mRNA vaccine also exhibited robust efficacy, offering a more translatable combination strategy for clinical application [197].

Concurrently, OVs show promise with conventional modalities: OVs combined with radiotherapy (RT) convert immunologically “cold” tumors into “hot” ones, supporting the success of triple therapy (OV, RT, and an ICI) in ICI-resistant skin cancer [198].

For chemotherapy, a targeted co-delivery strategy uses nanoscale carriers to deliver both an oncolytic measles virus (OMV) and vincristine (VC), enhancing efficacy while reducing chemotherapy dosage in prostate cancer [199]. Finally, combining OVs with targeted therapies is a rational strategy, as demonstrated by the initiated Phase III trial combining the OV Pexa-Vec with the kinase inhibitor sorafenib, capitalizing on the synergy between virotherapy and kinase inhibition [200].

Despite these advancements, the clinical efficacy of combination therapy is not yet broad across all tumor types and remains patient-variant. Future progress will require not only rational partner selection but also precise coordination of biological timing, delivery strategy, and immune context. Mechanistically informed trial designs incorporating sequencing variables and biomarker-guided stratification may ultimately determine whether OV-based combinations achieve durable and broadly applicable clinical benefit.

## Conclusion: future prospects and challenges of oncolytic virotherapy

Oncolytic virus therapy, as a novel cancer treatment modality combining direct oncolysis and immune activation functions, has demonstrated clinical potential in solid tumors such as melanoma and GBM. This article systematically integrates research, elucidating the core mechanisms of OVs, diverse viral platforms, clinical progress, and combination therapies with OVs. It focuses on analyzing innovative solutions through delivery, such as capsid modification and cell-mediated delivery, which can specifically address part of bottlenecks.

Despite significant progress in OV therapy, clinical translation still faces four core challenges. These issues are interrelated and collectively constrain efficacy: poor targeting leads to drug waste and off-target toxicity, immune clearance limits viral survival time, insufficient replication weakens the oncolytic effect, and resistance leads to treatment failure.

Building upon the existing body of research pioneered by previous scientists, it is imperative to continue advancing these foundational efforts while actively striving to enhance the efficacy of OVs across multiple dimensions. Furthermore, future investigations should address current research gaps and expand into the following three key areas:

1. Personalized OV therapy: Based on patient-derived models (e.g., PDOs [201]) and multi-omics analysis, screening patient subgroups suitable for OVs and designing personalized “OV + combination therapy” regimens;
2. Safety and controllability optimization: Developing more precise “safety switches” (e.g., dual promoter regulation [28]), or reducing off-target mutations in OVs through base editing [202], to lower toxicity risks;
3. Improving clinical translation efficiency: Establishing universal evaluation standards for OV efficacy and safety, promoting multi-center clinical trials, and simultaneously developing non-invasive monitoring technologies (e.g., liquid biopsy [203]) to enhance clinical management efficiency.

With ongoing innovations in multiple technologies and the continuous accumulation of clinical evidence, OV therapy is poised to become an integral component of precision cancer immunotherapy, offering novel therapeutic options and renewed hope for patients with refractory malignancies and metastatic cancer.

## Data Availability

No datasets were generated or analysed during the current study.
